# Leaves Antimicrobial Activity of *Glycyrrhiza glabra* L. 

**Published:** 2010

**Authors:** Mahboubeh Irani, Marziyeh Sarmadi, Françoise Bernard, Gholam Hossein Ebrahimi pour, Hossein Shaker Bazarnov

**Affiliations:** *Faculty of Biological Sciences, Shahid Beheshti University, Teheran, Iran.*

**Keywords:** *Glycyrrhiza glabra*, Antimicrobial activity, Licorice, *Candida albicans*

## Abstract

Licorice (*Glycyrrhiza glabra *L.) is an important medicinal plant. In this study, the antimicrobial activities of ethanolic and aqueous extracts from licorice leaves were studied compared to root extracts activities. *Bacillus subtilis, Enterococcus faecalis, Klebsiella pneumoniae, Pseudomonas aeruginosa, Staphylococcus aureus *and *Escherichia coli*, and *Candida albicans *were used as test organisms. Antimicrobial activity was tested by paper disc agar diffusion and serial dilution methods in order to determine minimum inhibitory concentration (MIC) and minimum bactericidal concentration (MBC). The root and leave extracts showed activity against *Candida albicans*, and tested gram-positive bacteria in a dose dependent manner. The ethanolic extract of the leaves was the most active extract against gram-positive bacteria. Its effectiveness against strains provides hope that it can serve as an alternative therapeutic agent.

## Introduction

Because of the side effects and resistance that pathogenic micro-organisms build against the antibiotics, much attention has been paid to extracts and biologically active compounds isolated from plant species used in herbal medicine. Medicinal plants may offer a natural and new source of antibacterial agents for use.

*Glycyrrhiza glabra *L. (Fabaceae) is a native of south-east Europe and south-west Asia, including Iran. *Glycyrrhiza glabra *L. (licorice), is a very sweet, moist, soothing herb with anti-inflammatory and expectorant properties, controls cough, and has hormonal effects. Moreover, it detoxifies and protects the liver. Medicinally, it is used internally for Addison’s disease, asthma, bronchitis, coughs, peptic ulcer, and arthritis (1). Antimicrobial activities of roots and rhizomes have been studied in previous researches, but there are a few reports about the effect of licorice leaves against microorganisms (2). 

In this study, we have investigated and compared the antimicrobial activities of different extracts from the leaves and roots of Licorice.

## Experimental

*Plant material*

Roots and leaves of *Glycyrrhiza glabra *L. were collected during July (2007) from Abadeh in Iran. This plant was identified and stored in the Herbarium of Biosciences Faculty**, **Shahid Beheshti University (voucher number 8600100).

*Preparation of plant extracts*

In order to prepare various extracts from different parts of *Glycyrrhiza glabra *L., leaves and roots were shade dried at room temperature and powdered by an electric blender. 50 g of each of the powdered materials were macerated with 500 mL of water or ethanol 80% for 24 h. All the extracts were concentrated by rotary vacuum evaporator. The condensed products were weighed and kept at 4 °C prior to test.

*Test microorganisms*

*Candida albicans *(ATCC10231) was used as the fungal tested organism and *Staphylococcus aureus *(ATCC 25923), *Bacillus subtilis *(ATCC 465), *Enterococcus faecalis *(ATCC 29737), *Escherichia coli *(ATCC 25922), *Pseudomonas aerouginosa *(ATCC 85327), and *Klebsiella pneumoniae *(ATCC 10031) were used as the bacterial tested organisms. For the suspension, 1 × 10^8^ cells/mL was prepared and compared to that of the 0.5 Mc Farland standard tube. 

*Antimicrobial assay*

Antimicrobial activity of the above mentioned extracts was determined using the paper disc agar diffusion method. Petri dishes containing the Muller Hinton agar were prepared and each plate was separately inoculated with different cultures of organisms by swabbing aseptically on the whole surface of the agar with cotton wool. The filter paper discs (6 mm diameter) were impregnated with 20 μL of each extract (4, 8 mg.disk-1). The discs were dried and placed aseptically at the plates. Disks injected with 20 μL of solvent were served as negative controls, and Chloramphenicol discs (30 μg) were used as positive control. The petri dishes were incubated at 37 ± 0.1°C for 20-24 h. At the end of period, the inhibition zones formed on the media were measured. The positive antimicrobial activity was read based on growth inhibition zone. Evaluation of the inhibitory properties was carried out in triplicates.

The Minimum Inhibitory Concentration (MIC) value of extracts was determined by serial dilution method (3). The extracts were diluted in a two-fold manner to make different concentrations. Serial dilutions of each extract were individually placed in tubes labeled 1 to 10. Tube 1 was filled with 2 mL of Muller Hinton Broth including the extract stock solution. Only 1 mL of the stock solution in tube 1 was transferred to tube 2 and diluted with 1 mL of Muller Hinton Broth. This procedure was repeated for solutions in tubes 2 to 10. Each tube was then filled with 1 mL Muller Hinton Broth including bacterial suspension. The resulting mixtures were incubated at 37 ± 0.1°C for 24 h. The solvents served as negative control. Turbidity was taken as an indication of growth, and the lowest concentration which remained clear was recorded as the relative minimum inhibitory concentration. This test was repeated in duplicated.

In order to determine Minimum Bactericidal Concentration (MBC) values, 100 μL of the content of the tubes with no turbidity, were cultured on the Muller Hinton agar medium and incubated at 37 ± 0.1°C for 24 h. This test was repeated in duplicates.

## Results and Discussion

Extraction of dried powder gave the following yields: leave ethanolic (5.4%), leave aqueous (4%), root ethanolic (10%) and root aqueous (9.6 %) extracts.

As shown in Table 1, the extracts of *Glycyrrhiza glabra *L. exhibited a strong activity against *C. albicans *and tested gram-positive bacteria (*Staphylococcus aureus*, *Bacillus subtilis*, *Enterococcus faecalis*), except aqueous extracts from root that showed no inhibition zone against *E. faecailis*. Results obtained from the controls indicated that solvents had no effect on the microorganisms. The relative antibacterial activity of ethanolic extract was higher than aqueous extract. 

**Table 1:** Antimicrobial activity of ethanolic and aqueous extracts from the roots and leaves of *Glycyrrhiza glabra *L.

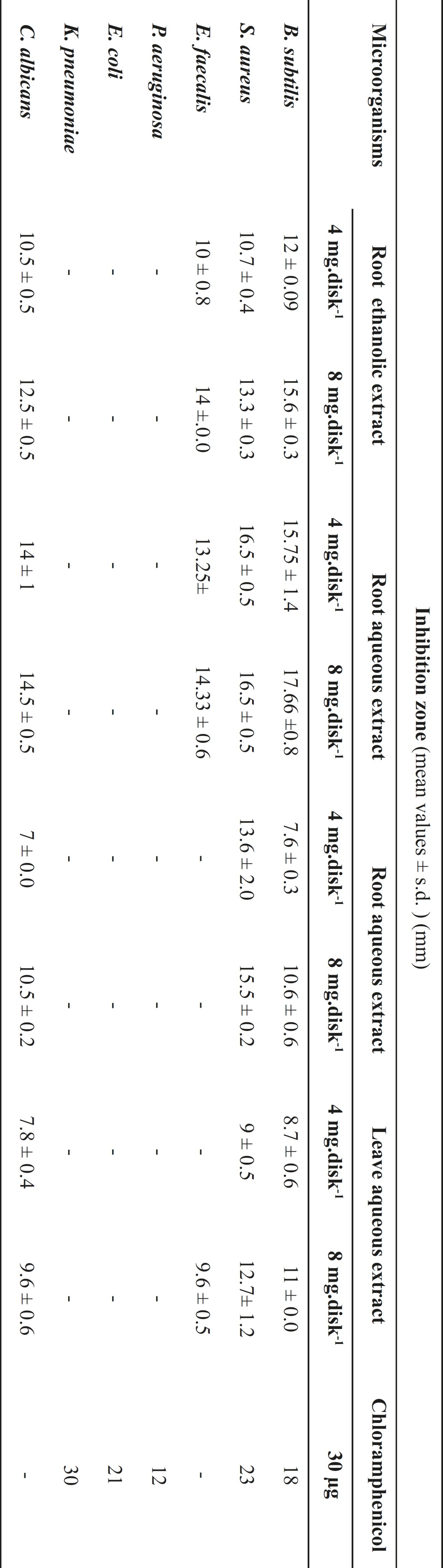

The MIC and MBC values are shown in Table 2. In most cases, leave extracts had better activity than root extracts. Ethnolic extracts from leaves of *G.glabra *showed the highest activity, whereas the value of aqueous root extracts against *C. albicans *was 625 μg/mL. This matter may be due to the materials extracted from various solvents.

**Table 2:** Minimal inhibitory concentrations and minimal bactericidal concentrations of various extracts of *Glycyrrhiza glabra *L. (mg.mL)

**Microorganisms**		Ethanolic extracts		Aqueous extracts	
		leave	root	leave	root
***Staphylococcus aureus***	MIC	1.25	2.5	10	5
	MBC	1.25	10	10	10
***Bacillus subtilis***	MIC	5	2.5	5	20
	MBC	10	5	20	40
***Enterococous faecalis***	MIC	0.312	5	10	-
	MBC	0.625	40	10	-
***Candida albicans***	MIC	5	2.5	0.625	1.25
	MBC	10	5	0.625	2.5

In the previous investigations, some results were reported, in which extract of licorice roots inhibit the growth of a gram-negative bacterium, namely *Helicobacter pylori *(4); while our results showed that leaves and roots extracts can inhibit the growth of some gram-positive bacteria. 

Ates *et al*. have reported various antibacterial activities (7-11 mm/20 μL inhibition zone) of the alcohol, acetone, and chloroform extracts of *Glycyrrhiza glabra *roots against the microorganisms tested; the alcohol extracts inhibit *B. cereus, K. pneumoniae, S. aureus*; the acetone extracts inhibit *B. cereus*, *B. subtilis, K. pneumoniae*, *S. aureus*; the chloroform extracts showed inhibition effect against *B. cereus*, *B. subtilis*, *E. faecalis*, *K. pneumoniae*, *S. aureus *(2). 

The majority of antimicrobial effects from licorice is due to isoflavonoid components particularly hispaglabridin and B,4’-O-methylglabridin, glabridin, glabriol and 3-hydroxyglabrol (5).

*S. aureus *is a major clinical pathogen. During the past decade, this bacterium has developed resistance to many commonly used antibiotics (6). In this study, the extracts of *Glycyrrhiza glabra *L. showed activity against *S. aureus *and can be used as raw materials for phytotherapy.

The present study provides further evidence about leaves antimicrobial activity of *G. glabra*.
